# Control trial of porcine cysticercosis in Uganda using a combination of the TSOL18 vaccination and oxfendazole

**DOI:** 10.1186/s40249-021-00823-6

**Published:** 2021-03-20

**Authors:** Zachary Nsadha, Chris Rutebarika, Chrisostom Ayebazibwe, Bukenya Aloys, M. Mwanja, E. Jane Poole, Elizabeth Chesang, Angela Colston, Meritxell Donadeu, Marshall W. Lightowlers

**Affiliations:** 1 College of Veterinary Medicine, Animal Resources and Biosecurity, Makerere University, P. O. Box 7062, Kampala, Uganda; 2 ANISOLUTIONS International Ltd, P.O. Box 610, Entebbe, Uganda; 3 Ministry of Agriculture, Animal Industry and Fisheries, Department of Animal Health, National Animal Disease Diagnostics and Epidemiology Centre (NADDEC), P.O Box 513, Entebbe, Uganda; 4 Malera Sub County Local Government, C/O P.O.BOX 5026, Bukedea, Uganda; 5 International Livestock Research Institute (ILRI), 30709, Nairobi, 0010 Kenya; 6 GALVmed, P.O Box 52773, Nairobi, 00100 Kenya; 7 Faculty of Veterinary and Agricultural Sciences, University of Melbourne, 250 Princes Highway, Werribee, VIC 3030 Australia; 8 Initiative for Neglected Animal Diseases (INAND), Midrand, South Africa

**Keywords:** *Taenia solium*, Pig, Porcine cysticercosis, TSOL18, Vaccination, Oxfendazole, Control

## Abstract

**Background:**

Neurocysticercosis caused by *Taenia solium* when the parasite lodges in the central nervous system, is an important cause of human seizures and mortality in sub-Saharan Africa. The parasite is prevalent in many regions of Uganda. Pigs are intermediate hosts for *T. solium*, and we evaluated a *T. solium* control program in pigs, involving vaccination of pigs with the TSOL18 vaccine and treatment with oxfendazole.

**Methods:**

The study was conducted in two districts of Eastern Uganda involving the rural village communities of Bukedea (intervention area) and Kumi (control area) during 2016–2017. Seven hundred and thirty-four households were enrolled in the study. Pigs in the intervention area received intramuscular immunizations with TSOL18 (Cysvax™) and an oral medication with 30 mg/kg oxfendazole (Paranthic™) at approximately 3-monthly intervals for 18 months. Porcine cysticercosis was evaluated by post-mortem examination. At the beginning of the study, 111 pigs were examined. In an interim evaluation in the intervention area, 55 pigs were evaluated 12 months after starting the project. At the end of the study approximately 3 months after the final intervention, 55 pigs from the intervention area and 56 pigs from the control area were evaluated.

**Results:**

The prevalence of porcine cysticercosis for the two sites was 16.2% at the beginning of the study (17.2% in the intervention area and 15.1% in the control area) with no statistically significant difference (*P* = 0.759) between the two study sites. Among the 110 animals assessed from the intervention site (55 at the interim evaluation and 55 at the final evaluation), no pig with viable *T. solium* cysts was found. There was a statistically significant difference between the prevalence at baseline (17.2%) and at the end of the study (0%) in the intervention area (*P* = 0.001) and a statistically significant difference between the intervention (0%) and control areas (5.4%) (*P* = 0.041) at the end of the study.

**Conclusions:**

Three-monthly concurrent vaccination of pigs with the TSOL18 vaccine and medication with oxfendazole eliminated *T. solium* transmission by the animals involved in the study. Application of vaccination with medication in pigs has the potential to reduce transmission of *T. solium* in Uganda and other endemic countries.

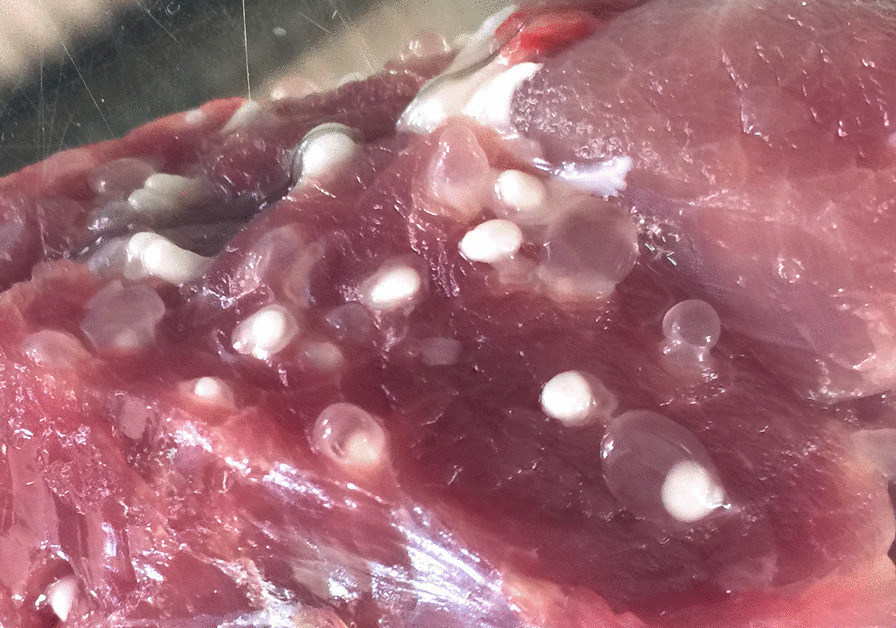

## Background

*Taenia solium* is an important cause of human morbidity and mortality in many countries in sub-Saharan Africa mainly due to neurocysticercosis, when the parasite encysts in the central nervous system [1–5]. Pigs act as the natural intermediate host for the parasite. Although there is substantial evidence for a high prevalence of porcine cysticercosis in many parts of Uganda [4, 6–8], there is very little data published about the prevalence of human neurocysticercosis in that country. Winkler and Richter [9] cite Winkler unpublished data as indicating a neurocysticercosis prevalence rate of 10% in persons with epilepsy living in rural areas of Uganda. Investigations in Uganda of subcutaneous lesions in onchocerciasis patients who suffered epilepsy were found in many cases to be caused by *T. solium* [10]. Alarakol and colleagues [11] found that up to 19.6% of epileptic patients in Uganda were serological positive for anti-*T. solium* antibodies. It seems likely that neurocysticercosis exacts a significant toll on human morbidity in Uganda, but that it is underreported.

There has been increasing interest in investigating the potential for control of transmission of *T. solium* by pigs through the combined use of the TSOL18 vaccine together with treatment at the time of vaccination with oxfendazole in order to eliminate parasites that may have established prior to vaccination [12, 13]. Field trials of this strategy have been successful in eliminating *T. solium* infection in pigs in Cameroon [14], Nepal [15] and Tanzania [16]. Programs incorporating pig vaccination and treatment, as well as treatment of the human population with taeniacide, have also been effective in eliminating *T. solium* transmission by pigs in a northern area of Peru [17] and in Zambia [18].

To date, there has been little attention paid to investigating potential strategies to reduce the transmission of *T. solium* in Uganda that could reduce the incidence of human neurocysticercosis. In order to assess the effectiveness of a vaccination strategy for reducing *T. solium* transmission through pigs in Uganda, we undertook a field evaluation of the TSOL18 vaccine in the Bukedea and Kumi districts of Eastern Uganda.

## Methods

### Study design

The study was conducted in rural village communities in Bukedea and Kumi districts of Eastern Uganda. Two distinct areas were involved, one a control area and the other an intervention area where pigs received concurrent vaccination with TSOL18 and medication with oxfendazole. Prior to undertaking baseline assessments of the prevalence of porcine cysticercosis, site surveys identified Bukedea and Kumi as suitable study areas, where pig owners were willing to consent to their pigs participating in the study. The study was undertaken in animals belonging to farmers who agreed to cooperate in the project. The owners and pigs were representative of the population of pigs of the study areas. Immediately prior to the trial beginning, healthy slaughter-age and weight pigs were randomly selected from pig owning households in the two study areas and subjected to post-mortem examination to determine the prevalence of porcine cysticercosis.

### Ethical approval

Conduct of the study was authorised by the appropriate National Regulatory Authorities in Uganda [the National Drug Authority (253/NDA/DID/08/15), the Uganda National Council for Science & Technology (A496), the Ministry of Agriculture Animal Industry and Fisheries and the Institutional Ethical Review Committee of the College of Veterinary Medicine, Makerere University, Uganda (VAB/REC/15/127)].

### Selection of study animals

The eligibility criteria for animals that were enrolled in the study were pigs > 8 weeks of age, not heavily pregnant and not clinically ill. In the treatment area, animals that were destined for slaughter within three weeks were excluded in compliance with the withholding period for the oxfendazole formulation that was used [19].

### Vaccination and pig medication

The day of first treatment administration to pigs was defined as Day 0. The intervention study site was visited at intervals of approximately 3 months and all pigs that met the inclusion criteria were enrolled continuously into the study, ear tagged, weighed, vaccinated and treated with oxfendazole. Animal weight in kilograms was estimated using a measuring tape (inches) and the formula [Girth^2^ × length/400]/2.2. The dose for oxfendazole (3 ml/10 kg Paranthic™ 10%, MCI Sante Animale, Morocco) was calculated according to the animal weight (30 mg/kg body weight) and was applied per os. Concurrently, 1 ml TSOL18 vaccine (150 µg TSOL18 in mineral oil adjuvant; Cysvax™, Indian Immunologicals Limited, India) was administered intramuscularly in the left side of the neck behind the base of the ear, prior to release of the animal. TSOL18 vaccine and oxfendazole were administered on Days 0 to 15 (first intervention), Days 106 to 118 (second intervention), Days 209 to 220 (third intervention), Days 308 to 318 (fourth intervention), Days 404 to 419 (fifth intervention), and Days 505 to 518 (sixth intervention).

### Assessment of pig infections

Infection with *T. solium* was determined at necropsy. The study was designed to be able to identify an 80% reduction in the prevalence of porcine cysticercosis in slaughter-age and weight pigs.

Sample size calculations were undertaken using a one-sided likelihood ratio test at the 5% significance level using SAS 9.3 (SAS Institute, Cary North Carolina, USA) with the TWOSAMPLEFREQ command in the PROC POWER procedure. Assuming an initial prevalence of infection of 20%, sample sizes of 55 animals were required in each area at the start and end of the trial in order to meet the desired statistical power. As a baseline, on Days -22 to -13, 58 slaughter-age pigs from the intervention area and 53 pigs from the control area were assessed. Approximately 3 months after the fourth intervention (on days 386–391) an interim evaluation of infection levels was undertaken in 55 animals that had received treatments on at least two occasions selected randomly from the intervention area. A final evaluation was made approximately 2–2.5 months after the final intervention involving a further 55 slaughter-age animals selected randomly from the intervention area, and 56 slaughter-age animals selected randomly from the control area on Days 572–580.

Post-mortem procedures were similar to those described by Sah et al. [20] and Poudel et al. [15]. The animals were transported to the National Animal Disease Diagnostics and Epidemiology Centre (NADDEC) Laboratories in Entebbe where they were euthanized by captive bolt pistol by experienced staff. The viscera were removed and the heart, liver, both kidneys and the full diaphragm retained in numbered containers. The carcase was skinned and divided cranio-caudally along the spine. The organs and the carcase, including the complete head, were refrigerated overnight at 4 °C. The head was removed and the tongue, masticatory muscles (both right and left sides) and brain were removed and retained. The muscles from the carcase were dissected from the bones.

### Examination for *Taenia solium* cysts

Except in cases of very heavy infection, all the retained organs and muscles of the right side of the carcase were sliced by hand at intervals of approximately 3 mm and examined meticulously for the presence of *T. solium* cysticerci or other lesions. During the necropsies undertaken on 111 animals at the end of the trial, when no cysticerci were detected in the tongue, masticatory muscles, diaphragm, brain or muscles from the right hand side of the carcase, the muscles of the left hand side of the carcase were also sliced. Cysticerci were counted and recorded as viable when they appeared as translucent vesicles filled with transparent fluid and having a visible white scolex. Non-viable lesions were recorded separately in cases where vesicles were non-translucent, containing a dense white or yellowish fluid and having no scolex and in cases of fibrosed or calcified lesions. Examples of suspect, non-viable lesions detected in organs other than muscle that were not calcified were placed into RNA-later (Sigma) and investigated by PCR analysis of a fragment of the mitochondrial 12S rDNA gene using the restriction enzymes *Dde*I and *Hinf*I or *Hpa*I, as described by Gauci et al. [21]. In carcases that contained thousands of cysts, all the heart, liver, kidneys, lungs, diaphragm, tongue, masticatory muscles and brain were sliced and counted and recorded as above. The remaining carcase musculature was weighed and representative samples from different muscle sites were selected representing approximately 1 kg. This sample was weighed accurately and then sliced and cysts counted as above and the number of cysts in the carcase muscles estimated from the total muscle weight.

### Definition of a case of confirmed porcine cysticercosis

The definition of a confirmed case of cysticercosis which was adopted by Sah et al. [20] was also used here. An animal was determined to be a confirmed case of porcine cysticercosis if one or more viable *T. solium* cysticerci was found in the muscle and or the brain, or if more than one non-viable lesion was detected in the muscles and/or brain. Animals having only non-viable lesions in organs that are not typical locations for *T. solium* (eg the liver, lungs or kidneys), and which could not be confirmed as being *T. solium* by DNA analyses, were excluded.

### Data analysis

Raw data was transcribed into pre-formatted Excel spreadsheets suitable for importation into the statistical software Genstat^®^ 18th edition (VSNi, registered in England and Wales). Results were summarised by treatment. Statistical analyses were undertaken to evaluate the effects of treatments on the prevalence of *T. solium* cysts at post-mortem examinations. Determination of the changes over a 24-month period in prevalence of *T. solium* cysts determined by carcass dissection of slaughter weight pigs in the intervention group were compared with the non-intervention control group. Prevalence of infection was determined before Day 0 and again after approximately 24 months. Results were compared within study treatment groups at baseline and end of study, using a two-sample binomial test. A generalised linear model with logit link function (logistic model) for binary data was used to compare results at baseline and endline between the groups and to provide standard error estimates and confidence intervals around prevalence figures (Genstat^®^ 18th edition). Prevalence data were based on cysts detected in the specified organs and muscles of the left-hand side of the carcase only. During endline necropsies, when no infection was detected in the organs or left side of the carcase, muscles of the right side were also sliced. Where any cysts were detected only in the right-side muscles, these data are presented descriptively, but were not included in the statistical comparisons.

## Results

### Intervention

Seven hundred and thirty-four households were enrolled in the study. Vaccination and medication were administered to 7535 pigs and a total of 12,204 doses of Cysvax vaccine and medication were used during the six interventions. Trial staff recorded no adverse effects in pigs after vaccination and medication. Overall, the number of pigs vaccinated and treated at each intervention remained approximately the same, averaging 2034 pigs (Table 1). All the pigs that met the inclusion criteria were vaccinated, however pigs that did not meet the inclusion criteria were not recorded so the coverage could not be determined. The average weight pigs of at each intervention varied between 16.9 and 24.0 kg over the six interventions (Table 1). The weight range was greater during the first three interventions (maximum 150 kg) compared with the last three interventions (maximum 95 kg).

**Table 1:** Numbers of pigs at each intervention and average weights

Intervention	Total pigs enrolled /intervention	Total pigs vaccinated /intervention	Animal weight		
			Average (kg)	Min	Max
1	1992	1992	16.9	3	125
2	851	1667	20.4	2	150
3	1116	2195	24.0	3	111
4	1387	2324	21.8	3	95
5	1296	2022	18.1	2	82
6	893	2004	20.1	4	72
Total	7535	12,204			
Average	1256	2034			

The number of total pigs vaccinated include pigs enrolled in previous interventions

The numbers of pigs which received 1, 2, 3, 4, 5, or 6 interventions during the trial is shown in Table 2. Forty-four percent of pigs had two or more vaccinations over the six interventions.

**Table 2:** Summary number and percentage of pigs receiving 1, 2, 3, 4, 5 or 6 interventions

No. of interventions received	Total no. of pigs	Percentage from total
1	4221	56.0
2	2011	26.7
3	838	11.1
4	336	4.5
5	110	1.5
6	19	0.3
Total	7535	

Numbers of pigs which received 1, 2, 3, 4, 5, or 6 interventions during the trial are shown in Table 2. Pigs would not have received all the treatments as there was a natural attrition due to sales and slaughter as the pigs reached the slaughter/sale weight.

The number of interventions received by each pig necropsied at the end of study post-mortems is shown in Table 3. Out of 55 pigs (29.1%) 16 had one intervention, 19 (34.6%) had two interventions, 13 (23.6%) had three interventions, three (5.5%) had four interventions, two (3.6%) had five interventions and two pigs (3.6%) had six interventions. The average weight of the intervention pigs was 25.4 ± 8.5 kg compared to 24.3 ± 7.8 kg for the control pigs, but the difference was not significantly different (*P* > 0.05).

**Table 3:** Intervention record of 55 treated pigs at end of study post-mortems

No. of interventions	No. of pigs	Inter1	Inter2	Inter 3	Inter 4	Inter 5	Inter 6	PC status
1	1	✓						Negative
	2				✓			Negative
	13					✓		Negative
2	6				✓	✓		Negative
	1				✓		✓	Negative
	12					✓	✓	Negative
3	1			✓	✓	✓		Negative
	12				✓	✓	✓	Negative
4	1	✓		✓	✓	✓		Negative
	2			✓	✓	✓	✓	Negative
5	2		✓	✓	✓	✓	✓	Negative
6	2	✓	✓	✓	✓	✓	✓	Negative

PC: Porcine cysticercosis as evaluated at post-mortem

### Prevalence and intensity of porcine cysticercosis

The prevalence of pigs infected with viable cysticerci at the three different times that necropsies were undertaken during the trial is shown in Table 4. During the baseline post-mortem evaluations 10 out of 58 (17.2%) pigs were positive from the intervention area and eight out of 53 (15.1%) pigs positive from the control area. There was no significant difference between prevalence in the two areas (*P* = 0.759) using the logistic regression model. The overall prevalence of porcine cysticercosis was 16.2%.

**Table 4:** Percentage prevalence of porcine cysticercosis in control and intervention areas

Study area	% Prevalence positive pigs (number of pigs)		
	Baseline	Interim	End
Intervention	17.2 (10/58)	0 (0/55)	0 (0/55)
Control	15.1 (8/53)	Nd	8.9 (5/56)^a^

Nd: Not done

^a^2 pigs out of the 5 had viable *T. solium* cysts in left side carcase and were not included in statistical comparison with baseline where the left side was not evaluated; 5.4% (3/56)

Of the 55 pigs necropsied from the intervention area during the interim evaluation, no animal was found to harbour viable *T. solium* infection. Similarly, at the end of the study none of the 56 pigs necropsied from the intervention area was found to have viable *T. solium* infection. In the control area five out of 56 pigs (8.9%) were found to have viable *T. solium* infection at endline, however, for two of these animals the cysts were only the right side of the carcase. At baseline the right side of the carcase was not evaluated therefore for statistical comparisons these two animals were not included, resulting in a prevalence for comparison of 5.4%. At endline there was a significant difference in prevalence in the two areas using the logistic regression model (*P* = 0.041).

There was a statistically significant difference between the prevalence at baseline (17.2%) and at the end of the study (0%) in the intervention area (*P* = 0.001) using the two-sample binomial test but non-significant difference between the baseline prevalence in the control area (15.1%) and endline (5.4%) (*P* = 0.092).

### Tissue distribution of cysts

The distribution of viable and non-viable cysts in the various tissues examined is shown in Table 5. No *T. solium* cysts were detected in the liver.

**Table 5:** Total count and percentage of viable and non-viable *Taenia solium* cysts in different tissue/carcass site of pigs

Organ/muscle	Baseline necropsy (*n* = 18)				End of study necropsy (*n* = 5)			
	No. V	Percent (%)	No. NV	Percent (%)	No. V	Percent	No. NV	Percent
Brain	69	0.10	12	0.02	3	0.20%	0	0.00%
Tongue	1109	1.56	725	1.25	20	1.33%	2	8.70%
Head/masseter	6300	8.85	2545	4.40	67	4.46%	0	0.00%
Forelimb	18 691	26.26	11 708	20.22	426	28.36%	20	89.96%
Flank	8743	12.29	15 020	25.94	-	-	-	-
Hindlimb	18 823	26.45	16 085	27.78	-	-	-	-
Heart	1795	2.52	831	1.44	14	0.93%	0	0.00%
Diaphragm	1586	2.23	468	0.81	52	3.46%	0	0.00%
Liver	0	0.00	0	0.00	0	0.00%	0	0.00%
Thorax	14 039	19.73	10 513	18.16	-	-	-	-
R.rest of carcass^a^					918	61.12%	0	0.00%
L.rest of carcass					2	0.13%	1	4.35%
Grand total	71 155	100.0	57 907	100.0	1502	100.0%	23	100.0%

It includes the 18 pigs positive at baseline, and the five pigs positive at end of study (including two pigs where a single viable cyst was found in the left side of the carcase musculature)

R.rest of carcass: Right side remainder of carcass musculature; L.rest of carcass: Left side remainder of carcass musculature; V: Viable; NV: Non-viable

^a^Flank, hind limb and thorax were all lumped into rest of carcass for the end of study necropsies

## Discussion

To our knowledge, this was the first evaluation of porcine cysticercosis levels in Ugandan pigs using a detailed post-mortem assessment. At baseline, 16% (18 out of 111) of slaughter-age pigs from the Bukedea and Kumi districts of Eastern Uganda were found to harbour viable infections with *T. solium*. A similarly high rate of infection was detected by Kisakye and Masaba [6] in pigs from the Lira district of central Uganda by carcase inspections which included incisions in various muscle areas. In animals coming from the southern Ugandan districts around Kampala, Kisakye and Masaba, the same authors [6] found no animals to be infected among 214 carcases that were examined. They suggested that the differences in prevalence were associated with animals free ranging in the Lira district but being either tethered or in pens in the districts around Kampala. In the districts where our trial was undertaken, approximately 60% of households allowed their pigs to roam freely, consistent with them potentially having access to human faeces and leading to the relatively high prevalence of porcine cysticercosis that we found. Several studies have used serological methods to assess porcine cysticercosis levels in Uganda [4, 7, 8], however these methods have been found to be unreliable due to cross-reactivity in the assays in pigs infected with *T. hydatigena* as well as high levels of positive reactions in pigs that have neither *T. hydatigena* nor *T. solium* infection [22–24].

Two assessments were made of animals in the intervention area after the introduction of vaccination and oxfendazole treatment, an interim assessment approximately 12 months after the 3-monthly interventions were implemented in the communities, and at the end of the trial 19 months after the interventions started. At both time points, no treated animal was found to harbour any viable *T. solium* cysts. This represented a statistically significant reduction in the prevalence of porcine cysticercosis in slaughter-age pigs, both in comparison to the prevalence in the intervention area at the start of the trial (*P* = 0.001), and in comparison to the prevalence of infection in the control area at the end of the trial (*P* = 0.041). There was no significant difference between baseline and end of study prevalence in the control area (*P* = 0.092).

Data have been published from field evaluations of TSOL18 vaccination of pigs together with oxfendazole treatment that were undertaken in Cameroon [14], Peru [17] and Nepal [15], Tanzania [16] and Zambia [18]. In each of these trials, *T. solium* transmission was eliminated by the pigs involved in the studies. The trials undertaken in Peru and Zambia also involved treatment of the human population with taeniacide. A further field trial tested a combination of the TSOL16 and TSOL18 antigens, achieved 99.9% protection [25]. The findings from our study undertaken in Uganda are consistent with the results from all previous trials in that combined use of the TSOL18 vaccine and oxfendazole eliminated the potential for *T. solium* transmission by the treated animals.

In the TSOL18 vaccine trials undertaken in Uganda and Nepal, treated pigs received both vaccination and oxfendazole treatment every 3 months. Animals sent for slaughter at about 1 year of age would have been expected to have received four treatments (e.g. at 2, 5, 8 and 11 months of age), with animals slaughtered at an older age receiving more treatments. A high frequency of treatments is required in order to ensure that new animals born into the population receive at least two vaccinations, together with at least one treatment with oxfendazole, by the time they are slaughtered for consumption.

A logical model of *T. solium* susceptibility in a pig population was developed by Lightowlers and Donadeu [13], with which vaccination and oxfendazole treatments were compared at different intervals for their effectiveness in prevention infected pigs transmitting the parasite. According to the model, 3-monthly treatments had the potential to prevent animals older than seven months from being able to transmit *T. solium*. Data accumulating from the field trials now completed in Uganda and Nepal, which adopted a 3-monthly treatment schedule, confirm the efficacy of this program of treatment to eliminate *T. solium* transmission by pigs.

While 3-monthly vaccination and medication of pigs may be effective in preventing porcine cysticercosis, a program involving the entire pig population may not be required in order to prevent porcine cysticercosis. Only two immunizations and a single treatment with oxfendazole are needed to provide protection to young animals [12]. In the trial conducted in Cameroon [14], vaccinated animals received three immunizations over a 12–13 month period. A pair of animals (one treated and one control) received TSOL18 vaccinations at the age of approximately 2–4 months of age (two injections four weeks apart) and a third injection approximately three months after the second injection protection assessed by necropsy 6–7 months after the third injection. Only a single oxfendazole treatment was given, at the time the animals received their second vaccination. While this was effective in eliminating the potential for *T. solium* infection in the treated animals, it involved a cohort of animals. It was not a program that could be applied to the whole pig population so as to ensure on-going treatments of new-born pigs, unless a mechanism was available to ensure vaccination and medication of young pigs in the communities on a continuing basis. Practical difficulties of implementing a control program in pigs in poor rural locations so as to ensure that that all young pigs received the required treatments, led us and others to deliver the treatments repeatedly to the whole pig population. While this approach has been successful in elimination infection in the pig population this needs to be fully costed and assessed against the benefits in reduction in the incidence human *T. solium* infection. The feasibility of implementing vaccination and medication of young pigs, only, in Uganda and other endemic countries should also be explored and evaluated alongside other control measures in order to bring about a sustained reduction in *T. solium* transmission leading to a reduction in the incidence of neurocysticercosis.

## Conclusions

Combined use of the TSOL18 vaccine and oxfendazole in pigs eliminated the potential for *T. solium* transmission in the animals involved in the trial. Future consideration needs to be given to a strategy that involves vaccination and treatments being given only to young animals in order to sustain an on-going *T. solium* control program.

## Data Availability

The datasets used and/or analysed during the current study are available from the corresponding author on reasonable request.

## Abbreviations

°C: Degree celsius
RNA: Ribonucleic acid
DNA: Deoxyribonucleic acid
rDNA: Ribosomal DNA
NADDEC: National Animal Disease Diagnostic and Epidemiology
PCR: Polymerase chain reaction
Min: Minimum
Max: Maximum
